# Mindful Parenting and Picky Eating Behaviors in Early Childhood: Parental Dietary Competence as a Mediator

**DOI:** 10.3390/children12121629

**Published:** 2025-12-01

**Authors:** Jo-Lin Chen, Su-Ping Chen, Jia-Yau Doong, Shou-Chi Huang

**Affiliations:** 1 Child and Family Studies, Fu Jen Catholic University, New Taipei City 242062, Taiwan; 046286@mail.fju.edu.tw; 2 Department of Living Services Industry, Tainan University of Technology, Tainan City 71002, Taiwan; t10061@mail.tut.edu.tw; 3 Nutritional Science Studies, Fu Jen Catholic University, New Taipei City 242062, Taiwan; 141600@mail.fju.edu.tw

**Keywords:** picky eating behaviors, healthy eating habits, parenting approaches, mindful parenting, parental dietary competence, early childhood

## Abstract

**Highlights:**

**What are the main findings?**

- The findings revealed that mindful parenting was indirectly associated with children’s picky eating behaviors through the full mediation of parental dietary competence.
- Both components of parental dietary competence—in dietary nutrition and in dietary regulation—serve as full mediators linking mindful parenting to lower picky eating behaviors.

**What are the implications of the main findings?**

- Strengthening parental dietary competence may be a key mechanism to translate mindful parenting into healthier eating behaviors among young children.
- Parenting programs that integrate mindfulness-based approaches with nutrition education may more effectively discourage picky eating and promote healthy habits in early childhood.

**Abstract:**

**Background:** Picky eating behaviors among children challenge nutritional intake and healthy development and place considerable stress on parents. Parenting approaches play a critical role in shaping young children’s dietary behaviors. Mindful parenting, which refers to parents responding to their child’s needs with awareness, acceptance, attention, and mindful discipline, has gained increasing attention. Parental dietary competence may determine whether mindful parenting effectively discourages picky eating behavior in children. **Objectives:** This study explored whether mindful parenting and parental dietary competence are associated with picky eating behaviors in young children. The mediating role of parental dietary competence in this association was also investigated. **Methods:** A total of 412 parents of children enrolled in six preschools across six administrative districts in New Taipei City, Taipei City, and Taoyuan City, Taiwan, were invited and recruited. Data were collected using three validated parent-reported instruments, including a mindful parenting scale, parental dietary competence scale, and children’s picky eating behaviors scale. **Results:** Mindful parenting influenced children’s picky eating behaviors through the full mediation of parental dietary competence. The identification of parental dietary competence as a mediator underscores the need for early preventive interventions and parenting education that integrate parental mindfulness and dietary competence to foster healthy eating habits from the outset of early childhood. **Conclusions:** Practical recommendations and future research directions are provided regarding mindful parenting, parental dietary competence, and picky eating behaviors in young children.

## 1. Introduction

Picky eating, characterized by food refusal and limited dietary variety, is common in early childhood. Prevalence estimates for picky eating range from 13.5% to 22% among children aged 2–10 years, underscoring its role as a major developmental concern [1,2]. Picky eating, often regarded as a normal developmental phase, has been linked to reduced dietary diversity and nutrient intake and increased family stress during mealtimes [3,4]. In a longitudinal study, Taylor and Emmett [2] demonstrated that picky eating first noted during preschool may persist into later childhood and adolescence, with children consuming fewer fruits and vegetables over time. These findings highlight the need for early picky eating recognition and intervention.

In addition to posing immediate dietary challenges, picky eating shapes physical and psychosocial health. Persistent refusal to consume certain food groups may lead to nutrient imbalances and inadequate dietary variety, which can disrupt normal growth and is associated with lower fruit and vegetable intake, as well as an increased likelihood of underweight among children [5,6]. In public health settings, picky eating should be considered alongside other problematic eating behaviors that contribute to adverse developmental and health outcomes [2]. Scholars should examine parenting-related factors that may contribute to preventing picky eating from persisting and that promote balanced and varied eating patterns from early childhood.

Mindful parenting refers to the integration of mindfulness principles into caregiving to promote intentional awareness, nonjudgmental acceptance, and regulation of both parent and child emotions [7,8]. The core elements of mindful parenting—intention, attention, and attitude—foster balanced and compassionate interactions [9]. Mindful parenting also strengthens emotional regulation and reflective functioning, enabling caregivers to better interpret children’s cues, reduce coercive feeding, and create a positive mealtime environment, minimizing conflict and facilitating harmonious parent–child interactions [10,11].

Greater engagement in mindful parenting has been linked to better emotional regulation, reduced reactivity, and more supportive parent–child interactions, fostering a healthy family climate and reducing parental stress [12,13]. In feeding contexts, mindful parenting has been associated with more responsive practices and less reliance on coercive strategies, such as pressuring children to eat or implementing food restrictions. This approach supports children’s self-regulation of hunger and satiety cues, thereby potentially limiting the persistence of picky eating [14]. Few scholars have directly examined the role of mindful parenting in shaping picky eating behaviors in early childhood; research in this area is warranted.

Feeding young children can be challenging; parental competence in this domain has been highlighted as an important factor shaping the dietary development of children [15,16]. Parental dietary competence refers to parents’ nutritional knowledge, feeding skills, and confidence when guiding children’s eating. Parental dietary competence corresponds to Satter’s [17] concept of eating competence, encompassing positive attitudes toward food, acceptance of dietary variety, internal regulation of intake, and contextual mealtime skills. Parents with greater dietary competence are better positioned to provide balanced meals and model healthy eating patterns for their children [18].

Parental dietary competence also encompasses practical regulatory skills, such as the ability to structure mealtimes and promote repeated food exposure through positive reinforcement [19]. The development of these competencies through targeted nutrition education programs can increase parental feeding self-efficacy and improve children’s food acceptance and dietary quality [20]. Empirical evidence has suggested that higher parental dietary competence is associated with healthier dietary intake among children, including greater consumption of fruits and vegetables and reduced intake of sugary foods [2,21]. Parents with higher dietary competence also tend to use consistent, responsive, and noncoercive feeding practices, which are linked to fewer eating difficulties and a lower risk of picky eating in young children [22,23].

Nutrition education and intervention studies have indicated that parental dietary competence can be strengthened to improve feeding strategies and children’s dietary quality, particularly in early childhood [24,25]. Few scholars have examined how dietary competence functions in tandem with parenting orientations, such as mindful parenting, to influence picky eating behaviors. Research that addresses this gap may inform holistic and culturally sensitive interventions for picky eating.

Despite growing evidence linking parenting styles and feeding practices to children’s eating behaviors, psychological orientations (e.g., mindful parenting) and skill-based constructs (e.g., parental dietary competence) have seldom been examined in relation to picky eating. This gap is particularly salient in Asian cultural contexts, where authoritarian feeding practices and beliefs, such as “eating more means being healthier,” remain prevalent [26,27]. Research on this topic has primarily focused on school-aged children or adolescents, neglecting preschool-aged children, whose eating behaviors are highly malleable [28]. This gap must be addressed to further scholarly understanding of how culturally rooted parenting practices influence the persistence of picky eating.

The present study investigated associations among mindful parenting, parental dietary competence, and picky eating behaviors in preschool-aged children in Taiwan, with a focus on the mediating role of parental dietary competence. The simultaneous exploration of these constructs within Taiwan’s cultural context can advance understanding of how parental awareness and dietary competence jointly shape early dietary development. Accordingly, whether parental dietary competence mediates the association between mindful parenting and picky eating behaviors in preschool-aged children was also tested.

## 2. Materials and Methods

### 2.1. Study Participants

This study targeted the parents of children aged 4–6 years enrolled in preschools located in New Taipei City, Taipei City, and Taoyuan City, Taiwan. The participating preschools were invited due to their willingness and capacity to implement the study procedures. A total of six preschools were included. Specifically, two urban administrative districts were selected from each municipality (one preschool from each district) to enhance sample representativeness. The research team clarified the types of assistance requested from preschools and provided consultation services and small gifts to express gratitude for their participation.

Recruitment sessions were conducted at the participating preschools, during which the research team explained the study to parents. Following these sessions, invitation letters were issued to formally invite parents to enroll in the study. The letters detailed the research objectives and procedures, the anonymous nature of responses, and the time required for participation. Parents were also given an opportunity to consult with the research team and ask questions to ensure that they fully understood the study procedures and their rights as participants.

Each preschool reported the number of parents interested in participating in the study. Questionnaires were subsequently distributed through the preschools. Completed questionnaires were returned in sealed envelopes to ensure confidentiality. To maintain participant privacy, the questionnaires were completed anonymously, and participants were identified using anonymized codes. Participants who completed and returned the questionnaire were eligible to receive a small gift. Completion and return of the anonymous questionnaire were considered to indicate the participant’s implied informed consent to take part in the study. The Institutional Review Board (IRB) of Fu Jen Catholic University, New Taipei City, Taiwan, approved this study in 2025 (FJU-IRB approval no. C113065). The IRB also granted a waiver of informed consent on the grounds that the questionnaire survey was conducted anonymously.

A total of 420 questionnaires were distributed, and 412 completed questionnaires were deemed valid, corresponding to a response rate of 98.10%.

### 2.2. Measurements

#### 2.2.1. Demographic Information

Demographic data, including parental age and educational attainment and child gender, date of birth, height, and weight, were collected. Parents reported their child’s height and weight based on the most recent measurements recorded by preschool staff. Child age was measured in months. Body mass index (BMI) values were calculated and classified in accordance with World Health Organization [29] Child Growth Standards as underweight, normal weight, overweight, or obese.

#### 2.2.2. Mindful Parenting Scale

The mindful parenting scale used in this study was originally developed by McCaffrey et al. [30], modified by Wu et al. [31], and further revised by Chen et al. [32] to suit parents of preschool children. Items were adapted to reflect parent–child mealtime situations. The adapted scale had 20 items across three subscales: awareness of oneself and one’s children (8 items), mindful discipline (7 items), and accepting and attentive interactions (5 items). The items were rated on a 5-point Likert-type scale with endpoints ranging from 1 to 5. Higher scores indicated greater engagement in mindful parenting. Exploratory factor analysis revealed a total explained variance of 54.42%, with item loadings within each subscale ranging from 0.43 to 0.76, indicating satisfactory convergent validity. Cronbach’s α values were 0.92 for the overall scale and 0.88, 0.86, and 0.70 for the awareness of oneself and one’s children, mindful discipline, and accepting and attentive interactions subscales, respectively. These values indicate that the scale was valid and reliable.

#### 2.2.3. Parental Dietary Competence Scale

Parental dietary competence was measured using an adapted version of the parenting competence scale developed by Chen et al. [33]. The adapted scale comprised 16 items across two dimensions: dietary nutrition (9 items) and dietary regulation (7 items). All items were rated on a 5-point Likert-type scale with endpoints ranging from 1 to 5. Higher scores indicated greater parental dietary competence. The analysis showed that this scale accounted for 64.10% of the total variance, with factor loadings for items across the two dimensions ranging from 0.47 to 0.81, suggesting adequate convergent validity. Cronbach’s α values were 0.94 for the overall scale and 0.92 and 0.90 for the dietary nutrition and regulation subscales, respectively, indicating that the scale was valid and reliable.

#### 2.2.4. Picky Eating Behavior Scale

Picky eating behaviors were evaluated using a scale developed by Chen et al. [23]. This scale comprised four items rated on a 5-point Likert-type scale with endpoints ranging from 1 to 5. Items with reverse wording were reverse-scored. Higher total scores indicated a higher frequency of picky eating behaviors among young children. The scale explained 64.38% of the total variance, and the factor loadings ranged from 0.72 to 0.85, providing additional evidence of strong convergent validity. Cronbach’s α value was 0.82, confirming the scale’s validity and reliability.

### 2.3. Statistical Analysis

SPSS Statistics version 29.0 (IBM Corp., Armonk, NY, USA) was used for all statistical analyses, including exploratory factor analysis, Cronbach’s α analysis, questionnaire item selection, descriptive statistics, correlation analysis, and mediation analysis using the PROCESS [34] macro. Parental and child demographic information and the distribution of the main study variables were summarized using descriptive statistics. Pearson product–moment correlation analyses were conducted to determine the associations between mindful parenting, parental dietary competence, and picky eating behaviors. The PROCESS [34] macro for SPSS was used to examine the mediating role of parental dietary competence in the association between mindful parenting and picky eating behaviors. Mediation effects were tested using 5000 bootstrap samples, and the significance of these effects was determined using 95% confidence intervals (CIs). For significant mediation effects, the PROCESS macro was applied to explore linear interaction effects across different percentiles of the mediator variables. The threshold for statistical significance was *p* < 0.05.

## 3. Results

### 3.1. Participant Characteristics

Among parents, mothers ranged in age from 25 to 49 years (mean [M]: 38.46 ± 4.50 years), and fathers ranged in age from 27 to 53 years (M: 40.41 ± 4.90 years). Regarding educational attainment, 280 mothers (68.0%) and 239 fathers (58.0%) had attained a university-level education (Table 1).

Among children, 209 (50.7%) were boys and 203 (49.3%) were girls. The average age was 67.92 ± 7.10 months. The average height was 112.41 ± 6.37 cm. The average weight was 19.81 ± 4.04 kg (range: 12–50 kg). In total, 313 children (76.0%) had a normal BMI, as classified in accordance with World Health Organization [29] Child Growth Standards. The remaining 34 (8.3%), 29 (7.0%), and 36 (8.7%) children were classified as underweight, overweight, and obese, respectively (Table 1).

### 3.2. Mindful Parenting, Parental Dietary Competence, and Children’s Picky Eating Behaviors

#### 3.2.1. Mindful Parenting

On average, parents frequently engaged in mindful parenting, with a total mean score of 4.05 out of 5. The mean scores by dimension were 4.20, 3.74, and 4.21 for awareness of oneself and one’s children, mindful discipline, and accepting and attentive interactions, respectively. Further details are provided in Table 2.

#### 3.2.2. Parental Dietary Competence

Parents demonstrated higher scores on the dietary regulation subscale (M = 4.00) than on the dietary nutrition subscale (M = 3.92). Scores were generally high on both subscales. Notable variations in specific items were observed. The highest- and lowest-scoring items in the dietary nutrition subscale were “Ensuring fresh and healthy foods are provided for children” (M = 4.13) and “Allowing children to participate in food preparation to increase their interest in food” (M = 3.53), respectively. In the dietary regulation subscale, the highest- and lowest-scoring items were “Guiding children to follow dietary rules” (M = 4.15) and “Avoiding the use of food as a reward or punishment” (M = 3.90), respectively (Table 3).

#### 3.2.3. Picky Eating Behaviors

After items with reverse wording were reverse-scored, the overall mean score was 2.64 ± 0.78, corresponding to occasional picky eating. The highest-scoring item was “My child is picky and eats only or refuses to eat specific types of food (excluding religious or allergy factors)” (M = 2.92 ± 1.00). The lowest-scoring item was “My child eats only foods prepared in a particular way” (M = 2.14 ± 1.00). Further details are provided in Table 4.

### 3.3. Correlations Between Mindful Parenting, Parental Dietary Competence, and Picky Eating Behaviors in Children

The three dimensions and the total mindful parenting score demonstrated weak but significant negative correlations with picky eating behaviors. In order of strongest to weakest, correlations were noted for mindful discipline (*r* = −0.23, *p* < 0.001), accepting and attentive interactions (*r* = −0.17, *p* < 0.001), awareness of oneself and one’s children (*r* = −0.16, *p* < 0.001), and total (*r* = −0.22, *p* < 0.001). Both dimensions of parental dietary competence likewise demonstrated weak but significant negative correlations with picky eating behaviors. Dietary regulation (*r* = −0.39, *p* < 0.001) demonstrated a stronger correlation than dietary nutrition (*r* = −0.36, *p* < 0.001). These results suggest that children are less likely to engage in picky eating behaviors if their parents practice mindful parenting and have a high level of parental dietary competence, and vice versa. Further details are provided in Table 5.

### 3.4. Mediation Analyses

Given the observation of significant correlations among the study variables, further analyses were conducted to determine whether parental dietary competence mediated the association between mindful parenting and picky eating behaviors. Preliminary analyses revealed no significant group differences across child age, gender, or parental education; therefore, these variables were not included as covariates in the mediation model and focused on the hypothesized relations.

The results of the mediation analyses are summarized in Table 6. Specifically, parental engagement in mindful parenting demonstrated a significant total effect on picky eating behaviors (b = −0.41, β = −0.22, *p* < 0.001, 95% CI: −0.58 to −0.23). At this stage, the mechanisms through which mindful parenting influences picky eating behaviors remained unclear, prompting further investigation. A parallel mediation model was constructed in which mindful parenting was specified as the predictor variable and the two dimensions of parental dietary competence (i.e., dietary nutrition and regulation) were tested as mediators in the causal pathway.

**Figure 1:**
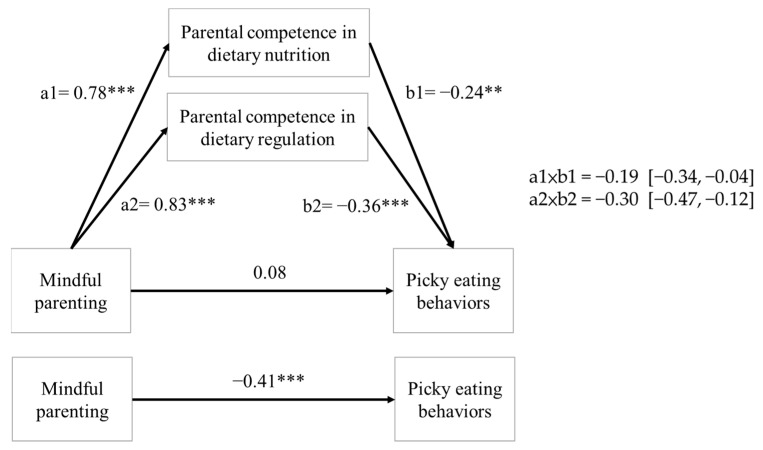
Mediating role of parental dietary competence in association between mindful parenting and picky eating behaviors. ** *p* < 0.01. *** *p* < 0.001.

The mediation analysis results indicated that mindful parenting indirectly influenced picky eating behaviors through parental dietary competence regarding both dietary nutrition (indirect effect: −0.19, 95% CI: −0.34 to −0.04) and regulation (indirect effect: −0.30, 95% CI: −0.47 to −0.12). By contrast, the direct effect of mindful parenting on picky eating behaviors was nonsignificant (b = 0.08, β = 0.04, *p* > 0.05, 95% CI: −0.13 to 0.28), indicating that both dimensions of parental dietary competence functioned as full mediators, as illustrated in Figure 1.

## 4. Discussion

This study examined the relations among mindful parenting, parental dietary competence, and picky eating behaviors in preschool-aged children in Taiwan. The findings revealed that mindful parenting was indirectly associated with children’s picky eating behaviors through the full mediation of parental dietary competence. These results underscore the importance of integrating mindful parenting and parental dietary competence in early childhood to promote healthy eating behaviors and prevent the persistence of picky eating behaviors. The following discussion elaborates on these findings in relation to previous research and highlights their practical implications.

Based on the descriptive results, children were rated as “sometimes” engaging in picky eating behaviors. In the study by Chen et al. [23], children “seldom” engaged in picky eating behaviors. The items “My child is picky and eats only or refuses to eat specific types of food (excluding religious or allergy factors)” and “My child refuses to eat new foods (i.e., those not tried before)” received higher mean scores in the present study than in the study by Chen et al. [23], indicating a stronger tendency among children in this sample to show food selectivity and avoid unfamiliar foods. Higher scores than those reported in the literature were noted for the item “My child eats only foods prepared in a particular way,” indicating a narrow acceptance of food preparation methods and a tendency toward rigid eating behaviors. These findings align with those of other studies on the characteristics of picky eating behaviors, highlighting how these behaviors may contribute to feeding challenges and parent–child conflict during mealtimes.

Consistent with other research [2,3], this study confirmed that picky eating is prevalent among preschool-aged children. Longitudinal evidence has highlighted the potential for picky eating behaviors to persist. For instance, children identified as picky eaters at age 3.5 years later consumed substantially fewer fruits and vegetables than their non-picky counterparts at ages 10 and 13, suggesting that early eating difficulties may shape dietary patterns into adolescence [35]. Picky eating during early childhood may substantially contribute to food waste. Studies have indicated that over 30% of school lunches are often discarded [36]. Such waste contradicts the United Nations Sustainable Development Goal (SDG) 12.3, which aims to halve global food waste by 2030 [37]. Addressing picky eating is therefore essential not only for promoting children’s health but also for advancing sustainability objectives. In particular, the early identification of picky eating is crucial, allowing for targeted parenting-related mechanisms to reduce long-term consequences. Collectively, this evidence provides a foundation for the examination of how parenting factors, such as mindful parenting and parental dietary competence, shape picky eating behaviors.

Turning to parental factors, parents in this study scored relatively high on both subscales of parental dietary competence, suggesting adequate nutritional competence and regulatory skills. Parental competence is particularly crucial in early childhood, during which children’s eating behaviors and habits are established [38]. Parents equipped with sufficient dietary competence are better positioned to foster a balanced and varied diet, thereby decreasing selective eating and promoting children’s dietary development and overall health [39]. Despite these strengths, certain aspects of parental competence appeared less well developed [40].

At a more detailed level, item-level analyses highlighted areas of comparatively low competence within the study sample. Within the dietary nutrition dimension, parents scored relatively low on the item “Allowing children to participate in food preparation to increase their interest in food,” possibly reflecting parental concerns regarding time constraints or safety. These concerns may limit opportunities for young children to engage in food preparation, consequently reducing their exposure to a broader range of foods. In the dietary regulation dimension, parents scored relatively low on the item “Avoiding the use of food as a reward or punishment,” indicating that some parents may use food as a mechanism for behavioral control. This practice risks creating negative associations with food in children’s minds, contributing to the persistence of picky eating behaviors.

In addition, mindful parenting was included in our analysis to explore how psychological orientations shape the enactment of competence in feeding contexts. In this study, overall scores indicated that parents usually engaged in mindful parenting. High scores were noted for the dimensions of awareness of oneself and one’s children and for accepting and attentive interactions, suggesting that parents generally remained aware of their own and their child’s emotions and engaged responsively in daily interactions. Scores in the mindful discipline dimension were lower, indicating that parental engagement in mindful practices was more limited when setting boundaries or enforcing rules.

Furthermore, these results tentatively suggest that awareness and acceptance may facilitate parental sensitivity when addressing children’s eating-related concerns, whereas mindful discipline may require additional support for its practical application. This interpretation is consistent with previous evidence linking mindfulness to improved emotional regulation and parent–child interactions [12,13]. Future longitudinal research is warranted to investigate whether enhancing mindful discipline results in measurable improvements in parental dietary competence and child eating behaviors.

Based on the correlational findings, parents who reported higher mindful parenting and greater dietary competence tended to report fewer picky eating behaviors in their children. These associations provided an empirical basis for proceeding with the mediation analysis, as significant correlations among the key variables were necessary for examining indirect pathways.

The mediation results tentatively suggest that parental dietary competence may play a central role in determining whether mindful parenting reduces picky eating behaviors. In other words, without adequate parental dietary competence, mindful parenting may be insufficient to guide children toward healthier eating behaviors. The mediation model further suggests that mindful parenting may strengthen both the nutritional and regulatory components of parental dietary competence, which may, in turn, contribute to lowering picky eating tendencies. Collectively, these findings provide preliminary evidence for an indirect mechanism through which mindful parenting may influence children’s eating behaviors via enhanced parental dietary competence.

Finally, a key contribution of this study lies in the identification of a full mediation pathway. Mindful parenting influenced children’s picky eating behaviors indirectly through parental dietary competence regarding dietary nutrition and regulation. Other studies have reported a partial mediating effect, whereby mindful parenting exerts direct effects alongside indirect pathways [41]. The full mediation observed in this study may reflect cultural or sample-specific factors, which should be regarded as tentative explanations and warrant further examination in broader Chinese or cross-cultural samples. These differences highlight the role of cultural context in how parental competence mediates mindful parenting.

Taken together, the findings of this study tentatively suggest that mindful parenting and parental dietary competence collectively shape young children’s eating behaviors. This finding is consistent with evidence suggesting that parents with mindful awareness, self-regulation, and concrete dietary knowledge can effectively foster healthy eating behaviors in children [13,42]. Thus, parenting cannot be considered an independent predictor; rather, both mindful parenting and parental competence should be conceptually integrated in future theoretical frameworks. On a practical level, the results may inform parenting education and intervention programs that combine mindfulness-based approaches with nutrition education to more effectively reduce negative eating behaviors and promote healthy eating habits in early childhood.

This study has several limitations. First, the reliance on parent-reported questionnaires may have introduced reporting biases, including social desirability and shared-method variance. Second, the cross-sectional design precluded causal inference. Although the observed mediation effect was significant, inference of causality requires longitudinal or experimental designs that can better establish temporal precedence. Third, the sample was limited to parents with children at preschools in northern Taiwan and primarily reflected urban families with relatively high education levels, limiting the generalizability of the findings. The full mediation effect observed may reflect the relatively high dietary competence of participating parents, and replication of this study in more socioeconomically diverse populations is warranted.

In future longitudinal or intervention studies, scholars should examine whether strengthening mindful parenting and parental dietary competence yields lasting improvements in children’s eating behaviors. Particular attention should be given to the dietary regulation component of parental competence and to reducing the use of food as a tool for behavioral control, which may be especially critical for preventing the persistence of picky eating. Additionally, other potential mediators and moderators, including parental stress, child temperament, and family mealtime dynamics, could be explored. Finally, cross-cultural comparisons should be conducted to assess whether the pathways identified here are specific to Asian cultural contexts or apply more generally to broader populations.

## 5. Conclusions

This study deepens our understanding of the mechanisms underlying picky eating behaviors in preschool children. The findings revealed a full mediation pathway, indicating that mindful parenting was indirectly associated with children’s picky eating behaviors through parental dietary competence, particularly in dietary nutrition and regulation. However, these findings should be interpreted with caution due to the study’s cross-sectional design, reliance on parent-reported data, and relatively highly educated urban sample. Parental dietary competence has emerged as a key mechanism linking mindful parenting with children’s eating behaviors, suggesting a potential target for preventive interventions that promote healthy eating in early childhood. These findings underscore the need to view mindful parenting and parental dietary competence as linked rather than independent contributors to children’s dietary behaviors. Accordingly, parenting education and intervention programs may consider integrating mindfulness-based parenting approaches with nutrition education to strengthen parental dietary competence and discourage picky eating behaviors in young children. Efforts to address these aspects of parenting may be beneficial for reducing the persistence of picky eating and fostering healthy dietary habits in young children.

## Figures and Tables

**Table 1:** The characteristics of the participants.

Variables	*n* (%)
Characteristics of caregivers	
Caregivers’ gender	
Mother	265 (64.3)
Father	147 (35.7)
Caregivers’ age (year), mean ± SD	
Mother	38.46 ± 4.50 ^a^
Father	40.41 ± 4.90 ^a^
Education level of mother	
≤High school	60 (14.5)
University	280 (68.0)
Graduate school	72 (17.5)
Education level of father	
≤High school	76 (18.5)
University	239 (58.0)
Graduate school	97 (23.5)
Characteristics of children	
Gender	
Boy	209 (50.7)
Girl	203 (49.3)
Age (month), mean ± SD	67.92 ± 7.10 ^a^
48–60 months	83 (20.1)
61–72 months	329 (79.9)
Height (cm), mean ± SD	112.41 ± 6.37 ^a^
Weight (kg), mean ± SD	19.81 ± 4.04 ^a^
BMI	
Underweight	34 (8.3)
Normal weight	313 (76.0)
Overweight	29 (7.0)
Obesity	36 (8.7)

^a^ Data are presented as mean ± standard deviation.

**Table 2:** Mindful parenting scores.

Subscale	Item Description	Mean Score ^a^ (SD)
Mindful parenting		4.05 (0.43)
Awareness of self and children		4.20 (0.48)
Mindful discipline		3.74 (0.57)
Interacting with acceptance and full attention		4.21 (0.46)

^a^ Five-point Likert-type scale (1–5; 1 = never, 3 = sometimes, 5 = always).

**Table 3:** Parental dietary competence scores.

Subscale	Item Description	Mean Score ^a^ (SD)
Parental competence in dietary nutrition		3.92 (0.61)
	Understanding the nutritional needs of children at developmental stages.	3.84 (0.78)
	Providing an appropriate balanced diet based on the child’s developmental stage.	3.84 (0.80)
	Ensuring the provision of fresh and healthy foods for children.	4.13 (0.74)
	Paying attention to providing a variety of foods.	3.97 (0.77)
	Identifying children’s dietary problems.	4.01 (0.70)
	Recognizing and responding to children’s dietary needs.	4.07 (0.70)
	Creating a pleasant dining atmosphere and environment.	4.02 (0.71)
	Allowing children to participate in food preparation to enhance their interest in food.	3.53 (0.94)
	Modifying or choosing healthier cooking methods for children (e.g., boiling, steaming, and stir-frying).	3.89 (0.86)
Parental competence in dietary regulation		4.00 (0.60)
	Guiding children to establish good eating habits, such as having fixed mealtimes and seating arrangements.	3.94 (0.80)
	Establishing proper table manners for children to follow.	3.96 (0.81)
	Guiding children to manage their own eating behavior.	4.04 (0.72)
	Guiding children to follow dietary rules.	4.15 (0.68)
	Avoiding using food as a reward or punishment.	3.90 (0.83)
	Establishing appropriate boundaries between meals and snacks.	4.05 (0.76)
	Using strategies and practices for parent–child family mealtime.	3.92 (0.73)

^a^ Five-point Likert-type scale (1–5; 1 = fully incompetent, 3 = partly competent, 5 = fully competent).

**Table 4:** Picky eating behavior scores.

Subscale	Item Description	Mean Score ^a^ (SD)
Picky eating behaviors		2.64 (0.78)
	My child enjoys trying different types of food. (reverse scoring)	2.59 (0.92)
	My child is picky and eats only or refuses to eat specific types of food (excluding religious or allergy factors).	2.92 (1.00)
	My child refuses to eat new foods (i.e., those not tried before).	2.91 (0.99)
	My child eats only foods prepared in a particular way.	2.14 (1.00)

^a^ Five-point Likert-type scale (1–5; 1 = never, 3 = sometimes, 5 = always).

**Table 5:** Correlations between mindful parenting, parental dietary competence, and picky eating behaviors.

	01	02	03	04	05	06	07
01 Awareness of self and children	1						
02 Mindful discipline	0.58 ***	1					
03 Interacting with acceptance and full attention	0.62 ***	0.60 ***	1				
04 Mindful parenting total	0.85 ***	0.87 ***	0.85 ***	1			
05 Dietary nutrition	0.50 ***	0.44 ***	0.46 ***	0.55 ***	1		
06 Dietary regulation	0.53 ***	0.50 ***	0.47 ***	0.59 ***	0.73 ***	1	
07 Picky eating behaviors	−0.16 ***	−0.23 ***	−0.17 ***	−0.22 ***	−0.36 ***	−0.39 ***	1

*** *p* < 0.001.

**Table 6:** Summary of mediation analyses.

Paths	Effect (95% CI)
Mindful parenting → Picky eating behaviors	
Total effect	−0.41 (−0.58, −0.23)
Direct effect	0.08 (−0.13, 0.28)
Indirect effect (Mindful parenting → Parental dietary competence → Picky eating behaviors)	−0.48 (−0.63, −0.35)
Mindful parenting → Parental competence in dietary nutrition → Picky eating behaviors	−0.19 (−0.34, −0.04)
Mindful parenting → Parental competence in dietary regulation→ Picky eating behaviors	−0.30 (−0.47, −0.12)

## Data Availability

The original contributions presented in this study are included in the article. Further inquiries can be directed to the corresponding author.
